# Temperature-induced reorganisation of *Schistocephalus solidus* (Cestoda) proteome during the transition to the warm-blooded host

**DOI:** 10.1242/bio.058719

**Published:** 2021-11-17

**Authors:** Ekaterina V. Borvinskaya, Albina A. Kochneva, Polina B. Drozdova, Olga V. Balan, Victor G. Zgoda

**Affiliations:** 1 Institute of Biology, Irkutsk State University, 3 Lenin St, 664025 Irkutsk, Russia; 2 Institute of Biology, Karelian Research Centre of the Russian Academy of Sciences, 11 Pushkinskaya Street, 185910 Petrozavodsk, Karelia, Russia; 3 Department of Proteomic Research and Mass Spectrometry, Institute of Biomedical Chemistry (IBMC), 10 Pogodinskaya street, 119121 Moscow, Russia

**Keywords:** *Schistocephalus solidus*, Proteome, Parasite, Cestoda, Plerocercoid

## Abstract

The protein composition of the cestode *Schistocephalus solidus* was measured in an experiment simulating the trophic transmission of the parasite from a cold-blooded to a warm-blooded host. The first hour of host colonisation was studied in a model experiment, in which sticklebacks *Gasterosteus aculeatus* infected with *S. solidus* were heated at 40°C for 1 h. As a result, a decrease in the content of one tegument protein was detected in the plerocercoids of *S. solidus.* Sexual maturation of the parasites was initiated in an experiment where *S. solidus* larvae were taken from fish and cultured *in vitro* at 40°C for 48 h. Temperature-independent changes in the parasite proteome were investigated by incubating plerocercoids at 22°C for 48 h in culture medium. Analysis of the proteome allowed us to distinguish the temperature-induced genes of *S. solidus*, as well as to specify the molecular markers of the plerocercoid and adult worms. The main conclusion of the study is that the key enzymes of long-term metabolic changes (glycogen consumption, protein production, etc.) in parasites during colonisation of a warm-blooded host are induced by temperature.

## Abstract (precis)

**Summary:** The study focuses on the processes that determine the survival of parasites in warm-blooded animals and demonstrates the potential for the application of proteomics in veterinary medicine.

## INTRODUCTION

Cestodes are obligate parasites with a complex life cycle, during which they are trophically transmitted from one type of host to another, often undergoing dramatic changes in environmental conditions. Moreover, the stress associated with the new environment in the new host even stimulates transformation or growth of the parasites. To explain the amazing ecological plasticity of these parasites, it is of interest to study the mechanisms of rapid and slow adaptation of cestodes, especially the molecular composition and gene regulation of tapeworms in response to external stimuli.

The parasitic worm *Schistocephalus solidus* (Müller, 1776), due to its specific ontogeny, is a suitable model for studying the transition of cestodes from cold-blooded to warm-blooded hosts. The first intermediate host of *S. solidus* is a freshwater copepod, while the second is the cold-water three-spined stickleback *Gasterosteus aculeatus* (Linnaeus, 1758), which receives the parasite when it feeds on infected zooplankton. In the body cavity of sticklebacks, helminths develop into the second larval stage (plerocercoid), which actively grows and accumulates nutrients. A plerocercoid that has reached a weight of about 50 mg becomes infective and is able to survive in the definitive hosts, piscivorous birds (Tierney and Crompton, 1992). If the intermediate host is swallowed by a bird, the parasite in the avian intestines undergoes heating up to 40°C in several minutes. This sharp rise in the ambient temperature (by 20-40°C depending on water temperature) triggers maturation and gametogenesis in the parasite. Within approximately 36 h, the worms mature, fertilise and release eggs, and at the end are excreted with the bird faeces (Smyth, 1950; Hopkins and Smyth, 1951).

The transcription of *S. solidus* genes, which presumably provide the successful colonisation of a warm-blooded host, have been previously investigated by Hébert et al. using *in vitro* experiments (Hébert et al., 2017). Overexpression of certain genes has been reported either in larvae or in adult worms (the ‘molecular signature’ of the corresponding life stage). Gravid worms have been shown to enhance the expression of genes of the reproduction and redox homoeostasis pathways compared to infective plerocercoids. Considering the possible differences between the actual *S. solidus* protein and transcript profiles due to RNA and protein processing, it would be useful to confirm the transcriptomic data by direct measurement of the parasite proteins. It would also be interesting to investigate the molecular rearrangements that occur in the very first moments after the temperature rise, the key event that triggers the maturation of *S. solidus* in the definitive host.

**Fig. 1.**
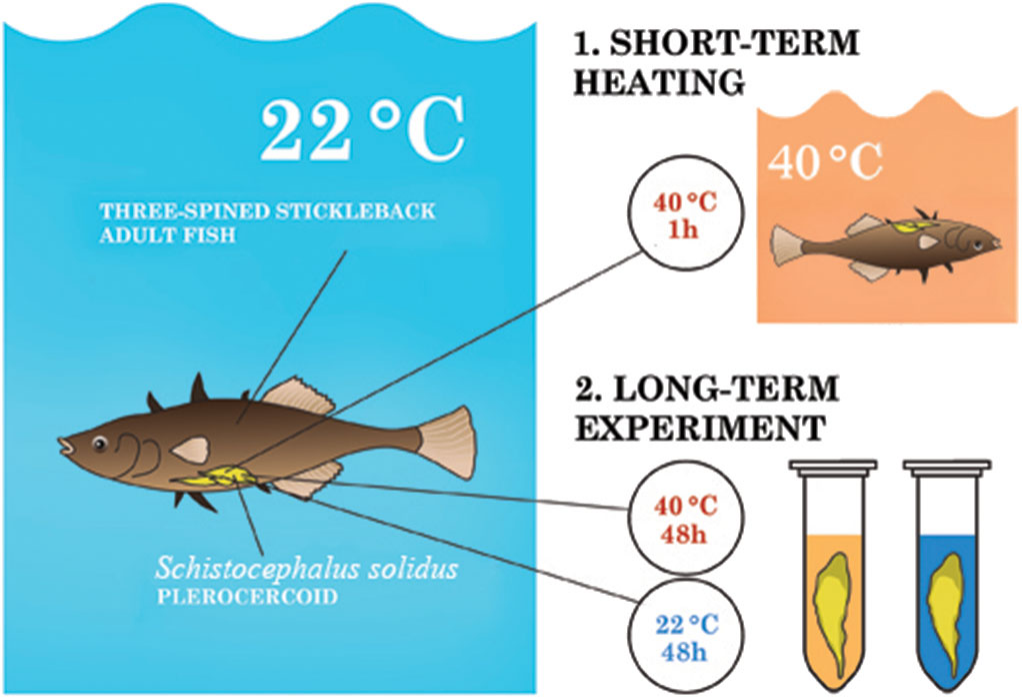
Experimental design.

Therefore, the aim of this study was to evaluate how the composition of proteins of *S. solidus* changes in an experiment simulating the transition of the parasite from fish to warm-blooded birds during short-term (1 h) and long-term (48 h) incubation of infective plerocercoids at 40°C (Fig 1). The main question was what molecular mechanisms ensure the adaptation of an infective parasite to a sharp increase in ambient temperature, and what genes are involved in the formation of the adult form of the parasite, as well as which of these genes are controlled by temperature.

## RESULTS

### Changes in protein profiles of *S. solidus* infective plerocercoids after short-term heating

The proteomic data obtained for worms incubated at 40°C for 1 h (*n*=3) inside the fish body without access to oxygen from the environment were compared with those of infective plerocercoids (*n*=3) taken directly from live fish kept at 22°C. This experiment was performed twice, in 2018 and 2019. The numbers of proteins identified in the extracts of heated and non-heated plerocercoids were 514 and 402 in 2018 and 2019, respectively; these two sets of proteins overlapped by 281 proteins (Table S4).

Multidimensional scaling analysis demonstrated a significant variability of the obtained data depending on the year of the experiment. This shift was probably caused by variations in the conditions of the chromatographic separation of the protein extract, despite following the same research protocol (Fig. S1). Thus, these two datasets were not combined but analysed for differential expression separately. In the 2018 and 2019 experiments, 151 and 13 proteins were differentially expressed in heated worms compared with not heated, respectively. Only one protein, A0A0X3PQ89, was identified as differentially expressed (downregulated upon heating) in both data sets. Thus, only the degradation of the A0A0X3PQ89 tegument protein was confirmed by repeated measurements in *S. solidus* plerocercoids during the first hour of heating.

### Changes in protein profiles of *S. solidus* infective plerocercoids after long-term heating

In the long-term experiment, after 48 h of incubation of *S. solidus* plerocercoids at 40°C, the worms began to release eggs into the media, indicating their maturation. In the control group, in which the worms were incubated at 22°C in the same environment, no egg production was observed.

**Fig. 2.**
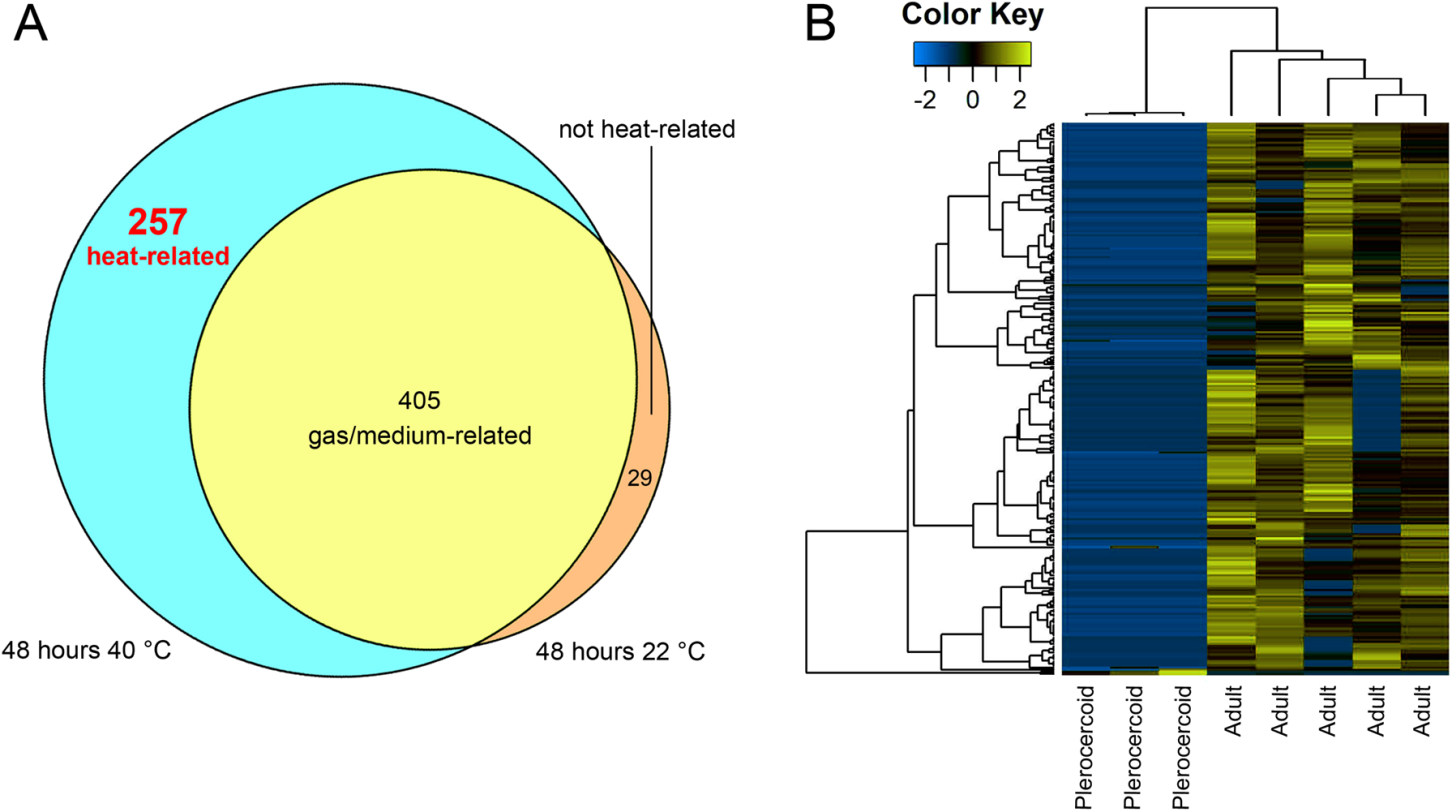
**Differential expression of *S. solidus* infective plerocercoids and adult worms.** (A) Venn diagrams represent the overlap of the lists of IDs of proteins differentially expressed between mature worms and infective plerocercoids. Blue indicates the number of proteins differentially expressed only in parasites incubated at 40°C for 48 h (‘heat-related’), while orange indicates proteins differentially expressed only in parasites incubated at 22°C for 48 h (‘not heat-related’), and yellow indicates proteins differentially expressed in both experiments (‘gas/medium-related’). (B) Heatmap of *S. solidus* ‘heat-related’ proteins, the level of which significantly changed only in worms that reached sexual maturity after incubation at 40°C for 48 h (for five biological replicates) in comparison with infective plerocercoids (for three biological replicates).

Since the stimulation of maturation was carried out in 2019, the proteomic composition of mature worms was compared with the proteome of infective plerocercoids obtained directly from fish in an experiment of the same year. After analysing the mass spectra of the incubated worms, 1361 *S. solidus* proteins were identified. Of these, 662 proteins of heated worms and 434 proteins of worms incubated at 22°C differed significantly in content compared to infective plerocercoids from the fish body cavity (Table S5). Notably, most of them (>98%) increased in amount after incubation at both 40 and at 22°C. The worms incubated at different temperatures shared 405 proteins with differential expression (Fig. 2A), and the concentration of all of them changed in the same direction.

**Fig. 3.**
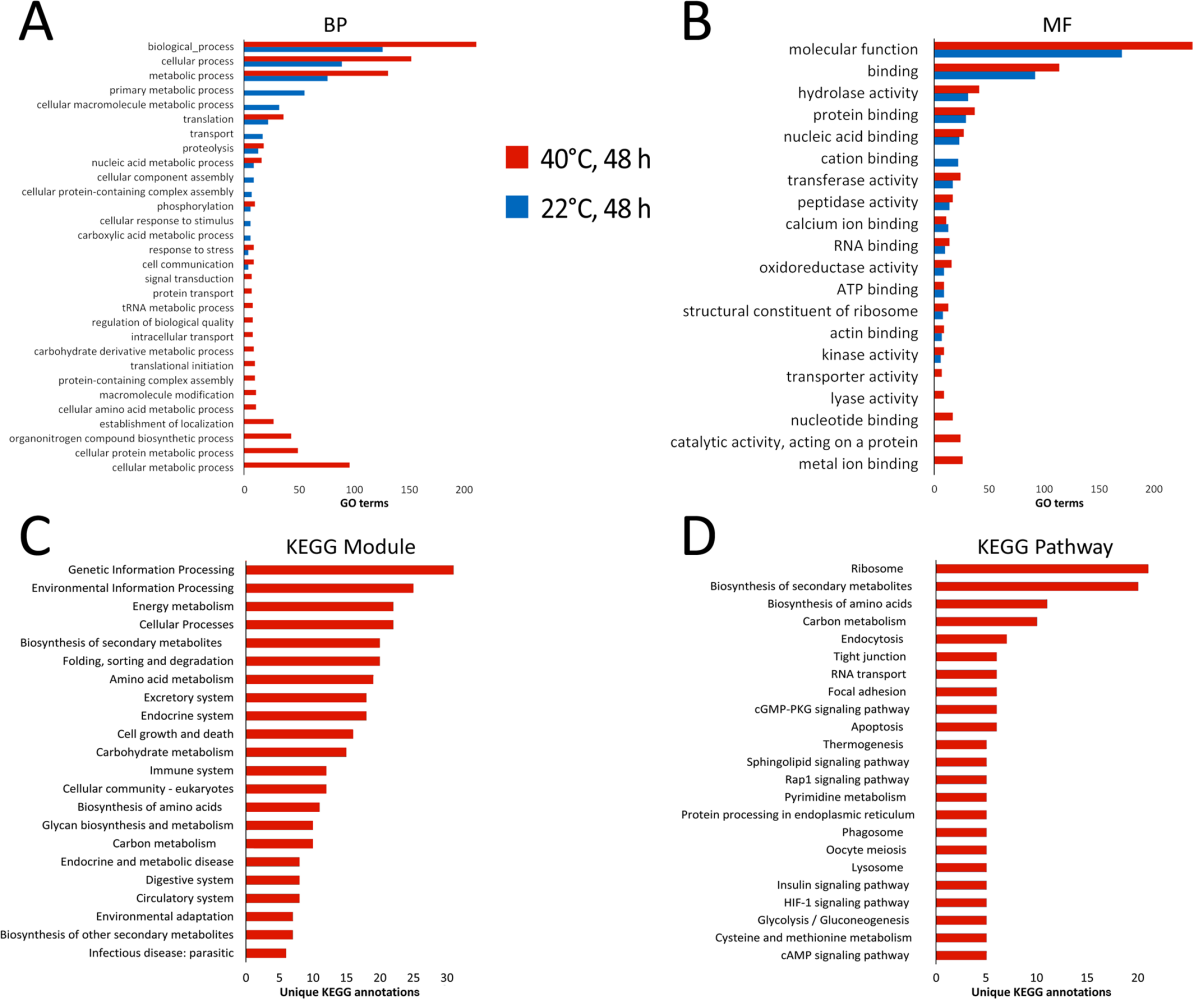
**Functional annotation of differentially expressed proteins (DEPs) in *S. solidus* cultured *in vitro* for 48 h at 40°C and 22°C in comparison with infective plerocercoids from live fish*.*** Note that *S. solidus* incubated at 22°C shared 93% of DEPs with parasites incubated at 40°C and that 99% of all DEPs were upregulated in mature worms compared to infective plerocercoids. (A) Top significant enriched GO terms of biological process ontology. (B) Top significant enriched GO terms of molecular function ontology. (C) Top KEGG modules of DEPs in adult worms that reached sexual maturity after incubation at 40°C for 48 h. (D) Top KEGG pathways of DEPs in adult worms that reached sexual maturity after incubation at 40°C for 48 h.

Enrichment analysis of differentially expressed proteins (Fig. 3A,B; Table S6) revealed that the parasites cultured at different temperatures shared most GO terms, although worms incubated at higher temperatures had more enriched terms for most processes. It should be noted that, in mature worms, the glycolytic process, electron transport chain, organonitrogen compound biosynthetic process, regulation of translation, vesicle-mediated transport, and other processes were specifically enriched (Table S6). Among proteins, the content of which differed between infective plerocercoids and mature worms, analysis of the KEGG ontology (Kanehisa, 2019) revealed a significant proportion of proteins involved in the processing of genetic information, energy production, carbohydrate metabolism, synthesis and processing of proteins (Fig. 3C,D).

## DISCUSSION

### Proteomic changes in *S. solidis* are mostly energy-related

During the rapid adaptation to a sharp increase in temperature in the definitive host, a decrease in the content of the platyhelminth-specific Tegument-Allergen-Like protein A0A0X3PQ89 (TAL, antigen Sm21.7) was observed in *S. solidus* plerocercoids after 1 h of exposure at 40°C under semi-anaerobic conditions (Table S4). Then, after 48 h of heating, the level of A0A0X3PQ89 in gravid *S. solidus* again became comparable to that in infective larvae (Table S5). TALs are known to be involved in the organisation and functioning of the tegument, but their biological role depends on the worm species and protein isoform (Fitzsimmons et al., 2012; Braschi et al., 2006; Zheng et al., 2013). For instance, all 13 members of *Schistosoma mansoni* TALs were shown to have different localisation and patterns of expression in adult and larvae parasites (Fitzsimmons et al., 2012). In *Echinococcus multilocularis,* similar to *S. solidus,* the A0A0X3PQ89 orthologue downregulates when metacestodes develop into pre-gravid adult worms, but gravid parasites again have high expression of the tegument protein (Zheng et al., 2013). We speculate that the initial decline in the content of tegument protein followed by recovery during worm maturation could be associated with tegument renovation occurring in the definitive host. At least in *S. solidus* plerocercoids, the initiation of massive cuticular peeling was described within an hour after ambient temperature increased to 40°C (Smyth, 1946). To elucidate the role of A0A0X3PQ89 in tegument loss or other adaptations at the early stages of final host colonisation, further study of the localisation of this protein is required.

The temperature increase triggers a maturation program encoded in the genomes of parasites, which launches a global restructuring of the protein profile and, as a result, changes the phenotype of the worm. In our long-term experiment, the infective *S. solidus* plerocercoids reached sexual maturity on the second day of exposure to the temperature of warm-blooded hosts, while the worms incubated at 22°C under the same conditions remained immature during this time. This is consistent with physiological and histological studies showing that temperature rise is a necessary factor triggering *S. solidus* meiosis and reproductive behaviour (Hopkins, 1950; Smyth, 1952; Schjørring, 2003).

It was found that in worms incubated *in vitro* for 2 days, about 600 proteins (almost half of the total observed proteome) had concentrations different from that of infective plerocercoids from the body cavity of live fish. Of these, up to two thirds change both at 40°C and 22°C (Fig. 2A), indicating that their synthesis is under the control of the composition of the culture medium (these proteins are hereinafter referred to as ‘gas/medium-related’). These proteins changed synchronously, i.e., they increased or decreased in content in both temperature modes (Table S5).

It is difficult to fully reproduce the internal environment of a living organism, especially considering that many parameters of *in vivo* systems are not fully understood. This study represents the first attempt to examine in detail the effect of temperature, one of the abiotic factors in a model system that mimics the colonisation of a warm-blooded host. However, with the current experimental design, it is still difficult to determine the leading factor in the culture medium that altered the expression of about a quarter of the proteins of the observed parasite proteome without heating. Therefore, it is unclear to what extent the observed changes are consistent with the natural process. Earlier, Smyth (1950), considering the extreme complexity of the internal environment of the avian intestines, proposed an approach according to which the exact reproduction of the ontogenesis of *S. solidus* matured *in vivo* by cultured worms (the same time of maturity, similar histological changes and hatching of normal embryo from produced eggs) indicates the absence of critical differences between artificial and natural media. According to this concept, maturing parasites are not very sensitive to the content of glucose, protein, lipids in the medium due to internal accumulated reserves (Hopkins, 1950), as well as to osmotic pressure, bile acids, hormones, and the specific microbial environment of the intestine, interaction with host immune cells and other factors essential for helminths with a long intestinal phase (Sinha, 1967; Frayha and Smyth, 1983). In turn, the pH of the medium and oxygen tension, along with temperature, are critical for maturation; therefore, their change can be a signal for the parasite that it has entered the internal environment of the definitive host (Smyth, 1956). Also, physical support, mimicking mechanical compression from the intestinal walls, is specifically important for the fertilisation process.

Enrichment analyses of differentially expressed proteins (Fig. 3A,B; Table S6) did not reveal modifications of specific metabolic pathways in our experiment, which could indicate the leading influence of some external stimulus on a prominent proteomic shift at 22°C. Among the proteins of the ‘response to stress’, a group of proteins involved in antioxidant defence can be distinguished, such as thioredoxin-like protein A0A0X3PKZ1, peroxidasin A0A0X3Q4L9, superoxide dismutase A0A0X3PDF0, glutathione peroxide A0A183SLN5, and peptide methionine sulfoxide reductase A0A183SZ65. The upregulation of these proteins may indicate an excess of oxygen in the media (this parameter was not controlled at the bottom of the test tube with the worm), although we did not find any enrichment in the pathways of response to hyperoxia or hypoxia. An increase in the expression of carbonic anhydrase subunits A0A0V0J4B5, A0A0X3PUJ7, A0A0V0JAB9 also indicates a possible effect of the gas composition and acid-base balance of the medium on the metabolism of the plerocercoid. At the same time, no enrichment of GO terms related to response to osmotic stress was found, although the content of some enzymes controlling the transfer of monovalent inorganic cations was increased, such as the subunits of Na^+^/K^+^-ATPase and the V-type proton ATPase (A0A0X3PRZ8, A0A0X3NTH8 and A0A0X3PDW9, A0A183SRG4, respectively), which could be a compensatory response to changes in pH or ionic composition of the medium. Thus, given the known limitations of artificial culture, further in this study we focused on the analysis of 257 proteins that were differentially expressed only in worms incubated at high temperature (hereinafter referred to as ‘heat-related’), as definitely associated with the adaptation of the parasite to a definitive host.

In our study, the number of such proteins that were different between adult *S. solidus* and infective plerocercoids was fewer than the number of differentially expressed genes in a similar experiment performed by Hébert and colleagues (2017). This can be explained by the lower sensitivity of proteomic methods compared to the analysis of the transcriptome, as well as the different effects of mRNA and/or protein processing in cells on the results of these two methods (Sun et al., 2014). It should also be noted that in the study by Hébert and colleagues, there was no control group to evaluate the effect of culture medium on the expression of the genes that may have a different transcription pattern in the avian intestine. Nevertheless, a comparison of independent transcriptomic and proteomic datasets revealed gene products that were differentially expressed in the same way in adult worms in both studies. From these, we filtered out 80 ‘heat-related’ genes whose expression is regulated by temperature, and which can be considered as reliable markers of the adult or plerocercoid stage (Table 1).

**Table 1.**
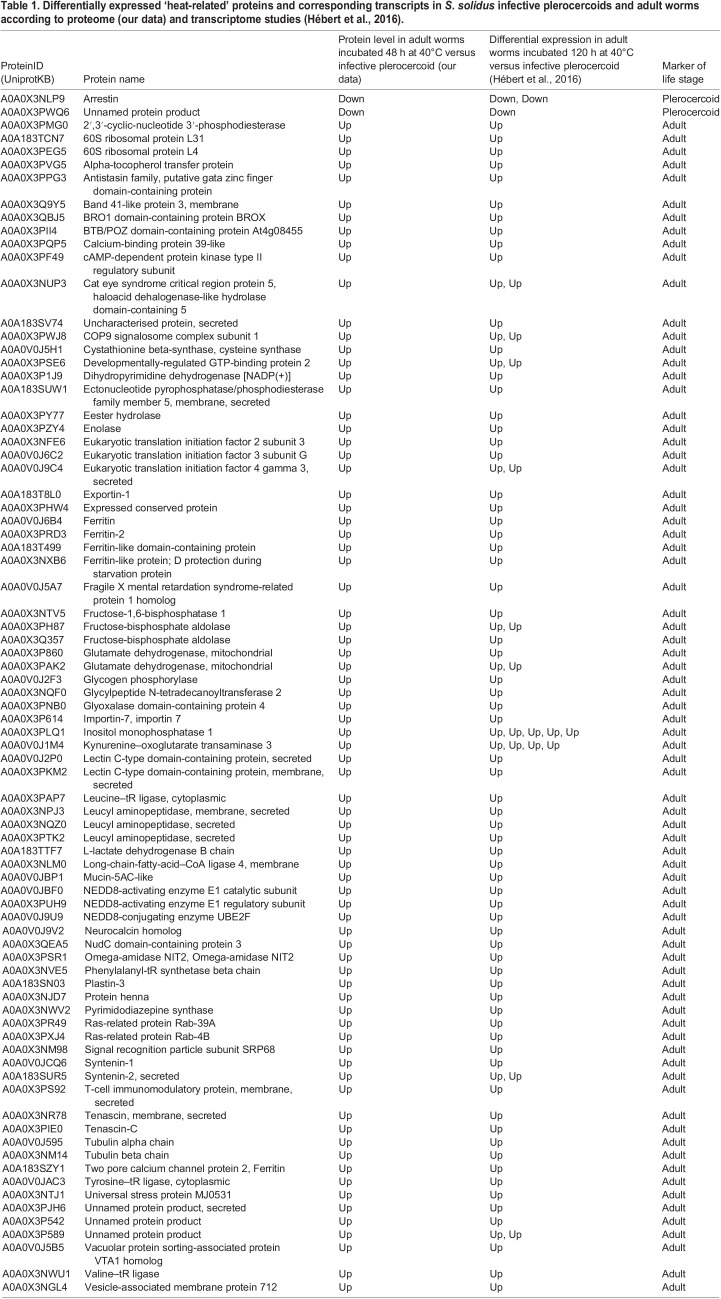
**Differentially expressed ‘heat-related’ proteins and corresponding transcripts in *S. solidus* infective plerocercoids and adult worms according to proteome (our data) and transcriptome studies (****Hébert et al., 2016****).**

According to the obtained proteomic data, the content of almost all heat-related proteins was increased in adult worms as compared to plerocercoids (Fig. 2B). This indicates global metabolic activation in maturing *S. solidus* after the dormant stage due to the need to complete the ontogenetic cycle in just a few days and convert the energy accumulated by the larvae into the maximum number of gametes and eggs. An intense rearrangement of the metabolism of adult parasites can also be seen due to the significant proportion of proteins involved in the processing of genetic information among heat-induced proteins (Fig. 3C).

Only two proteins, arrestin A0A0X3NLP9 and uncharacterised protein A0A0X3PWQ6, were more abundant in plerocercoids than in adult worms (Table 1). At the transcriptome level, down-regulation of at least five arrestin isoforms in adult worms was previously detected by Hébert with colleagues (2017) (Table S5). Moreover, the maximum expression of the arrestin isoform A0A0X3NLP9 was recorded in pre-infective plerocercoids, which did not yet gain sufficient mass (<50 mg), less in plerocercoids capable of infecting birds, and minimal in mature parasites, while the expression of the other four isoforms was maximal in infective plerocercoids. This indicates the great importance of arrestins, which are known to function in signal transduction, for the regulation of the second larval stage of *S. solidus*, although different isoforms regulate different periods of larval development. Sequence alignment of the uncharacterised protein A0A0X3PWQ6, suppressed in adult parasites, revealed orthologues in platyhelminths and a relationship with proteins of other organisms containing the Srp40 domain. This domain has been reported to be involved in ribosome processing, modification, and regulation of RNA polymerases; however, the significance of these proteins for flatworms remains to be elucidated.

Based on the analysis of the KEGG ontology and Gene Ontology enrichment analysis, the predominance of anabolic processes in the body of an adult worm can be stated, of which the pathways of protein synthesis and processing can be noted as the most affected (Fig. 3C,D; Tables S6 and S7). In mature worms, the level of 15 ribosome proteins, two proteins responsible for mRNA transport and splicing, one protein responsible for RNA degradation (RNA helicase DDX6), five translation initiators (components of translation initiation multifactor complex and eIF4G), one translation inhibitor (FMRP translational regulator factor) and two translation termination factors (eukaryotic peptide chain release factor, serine/threonine protein phosphatase 2A regulatory subunit) significantly increased (Figs S2 and S3; Table S7). Signal peptidase complex subunit 3, two subunits of signal recognition particle (SRP) and four subunits of proteasome, which presumably determine post-translation fate of newly synthesised proteins, were also found to be abundant in the proteome of the mature parasite.

In adult worms, the increased expression of enzymes that regulate the metabolism of tyrosine, tryptophan, alanine, serine, glutathione, cysteine, glutamine, putrescine, spermidine, selenohomocysteine, and others may indicate reinforced production of amino acids and regulators of protein synthesis. Glutamate dehydrogenase, a key nitrogen/carbon metabolism switching enzyme, was also elevated in mature worms. This enzyme provides the assimilation of ammonia in the form of glutamate or, conversely, releases α-ketoglutarate for the tricarboxylic acid cycle. Glutamate dehydrogenase has been found directly in trematodes vitellaria (Abidi et al., 2009), and its overexpression was observed in sexually mature parasites *S. solidus, Echinococcus granulosus*, *Opisthorchis felineus* and *Schistosoma mansoni* compared to their larvae and immature adults, indicating a crucial role in flatworm fertility (Protasio et al., 2012; Zheng et al., 2013; Hébert et al., 2017; Ershov et al., 2019). Interestingly, the *S. solidus* glutamate dehydrogenase isozyme A0A0X3P860 contains the GFGNVG sequence and Glu (278), which are more typical for isoforms that provide the deamination reaction rather than the addition of ammonia (Oliveira et al., 2012; Prakash et al., 2018; Grzechowiak et al., 2020). Therefore, in adult flatworms, this enzyme appears to provide NADPH for redox reactions, probably for the malate dismutation pathway in mitochondria (Smyth and McManus, 2007; Ritler et al., 2019).

The use of energy reserves accumulated by the larva through glycogen breakdown is initiated in adult *S. solidus* by the enhanced synthesis of glycogen phosphorylase A0A0V0J2F3 (Table 1) (Hopkins, 1952; Hébert et al., 2017). However, our study confirms that, in adult worms, only fructose-1,6-bisphosphatase, aldolase and enolase, but not the full set of glycolysis/gluconeogenesis enzymes, are activated (Table 1). Three out of five aldolase isoforms (A0A0X3PH87, A0A0X3Q357 and A0A0X3NM70) identified in the mass spectra of the parasite were found only in adult worms. Moreover, enolases were not detected at all in the transcriptome and proteome of the plerocercoids, while in adults, six isoforms of the enzyme were detected by LC-MS/MS. Fructose-1,6-bisphosphatase A0A0X3NTV5, which catalyses the only irreversible reaction of gluconeogenesis, was also absent in the proteome of larvae and present in adult worms, while the concentration of fructokinases catalysing the competitive reverse reaction did not change. These results are in general consistent with the study by Körting and Barrett (1977), who reported the presence of low aldolase and fructose-1,6-bisphosphatase and enolase activity in *S. solidus* plerocercoids compared to other glycolytic enzymes.

**Fig. 4.**
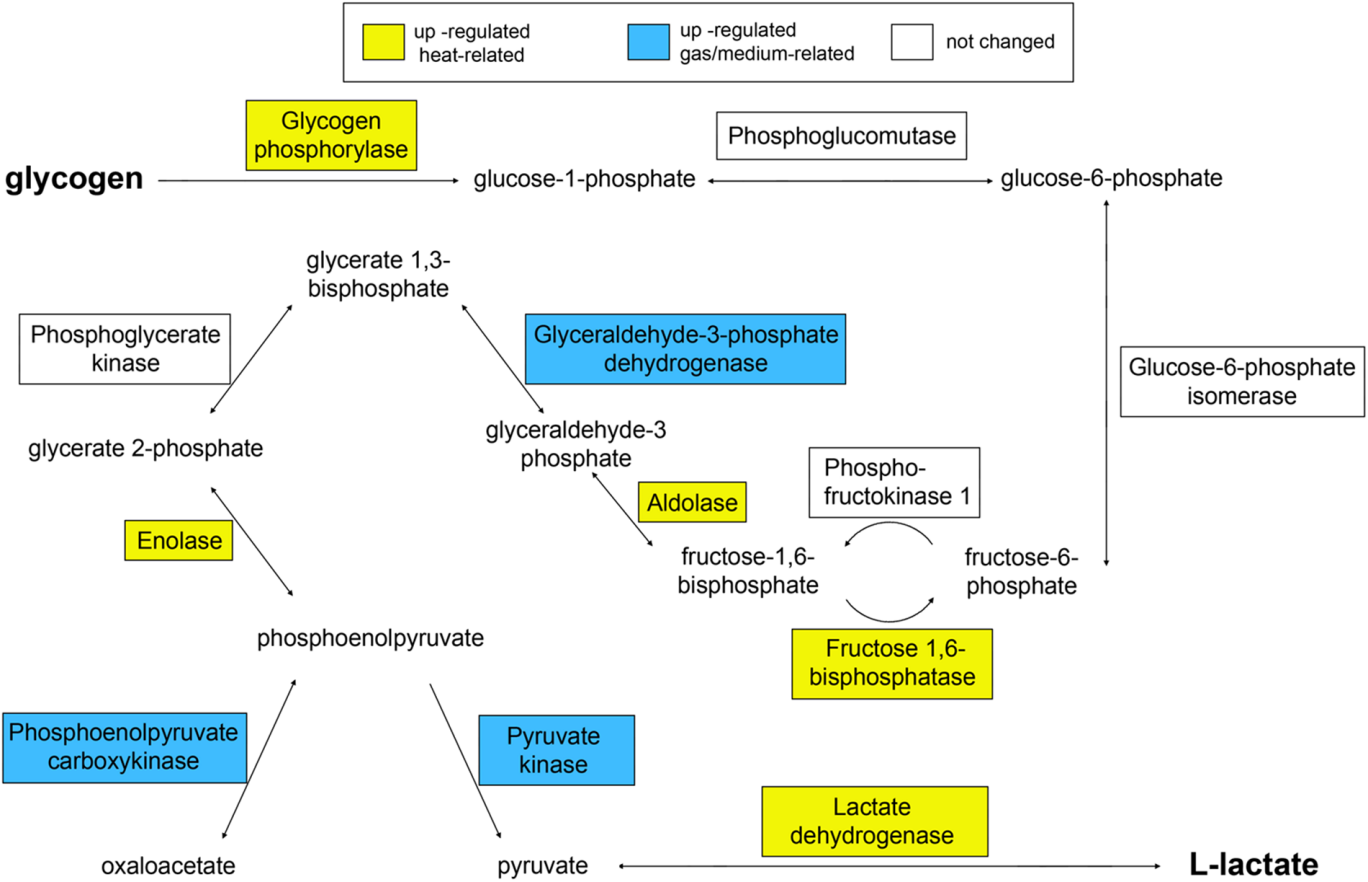
Scheme of glycolysis/gluconeogenesis pathway highlighting the proteins with increased concentration in adult worms compared with infective plerocercoids.

Earlier, Hébert et al. (2017) recorded a decrease in the level of transcripts of other glycolytic enzymes in adult worms, but according to proteomic data, their content remained at the same level as in the larva. The activity of phosphoglycerate kinase, glucose-6-phosphate isomerase and phosphoglucomutase was reported to be quite high in *S. solidus* plerocercoids (Körting and Barrett, 1977); therefore, glycolysis is possible in adult worms, but the carbon flux is most likely directed toward the production of glucose or glycolysis intermediates rather than the complete glucose breakdown (Fig. 4). We can also assume that overexpressed glycolytic enzymes play an alternative role in mature parasites, not necessarily associated with the metabolism of their own carbohydrates. For example, they can be synthesised for export, since glycolysis enzymes have been found in the secret of the marita of the trematode *Opisthorchis felineus*; schistosomules of the trematode *Schistosoma japonicum*; *Taenia solium* metacestode and an adult *Hymenolepis diminuta* (Victor et al., 2012; Lvova et al., 2014; Bień et al., 2016; Cao et al., 2016). It is known that enolase is a marker of exosomes, vesicles that detach from the parasite's cell membranes and deliver signalling molecules to their host (Buck et al., 2014; Samoil et al., 2018). In addition, various glycolytic enzymes are abundant in the shells and eggs of parasitic worms (Dewalick et al., 2011).

In cestodes, glucose oxidation leads either to the formation of lactate, or to the formation of oxaloacetate, and then malate, which is converted into succinate and acetate in mitochondria (CO_2_ fixation pathway; malate dismutation pathway) (Smyth and McManus, 2007; Ritler et al., 2019). This cleavage occurs at the level of phosphoenolpyruvate, for which pyruvate kinase (PK) and phosphoenolpyruvate carboxykinase (PEPCK) compete (Das et al., 2015) (Fig. 4). The level of PK A0A0X3Q057 and one of the PEPCK isoforms A0A183TAB4 was increased in worms kept at both 40°C and 22°C, which suggests that the expression of these enzymes can be controlled by the composition of the medium. In turn, pyruvate formed by PK is converted by lactate dehydrogenase to lactate, which is the end product of *S. solidus* metabolism in a warm-blooded host (Smyth and McManus, 2007; Beis and Barrett, 1979). We found that two of the three isoforms of lactate dehydrogenases (A0A183TTF7, A0A3P7E2D4) identified in the mass spectra of the parasite were present only in adult worm proteomes. This enzyme promotes energy production when there is a lack of oxygen in the intestines of birds. Our study confirms the conclusion of Hébert and colleagues (2017) that the synthesis of enolase and some isoforms of aldolase, fructose-1,6-bisphosphatase and lactate dehydrogenase is a phenotypic marker of the sexually mature stage of *S. solidus* (ecological status of the annotation ‘switched ON in the bird’), since these enzymes are under the control of genes that are induced by an increase in temperature. In turn, the fate of the glycolysis product, phosphoenolpyruvate, is controlled by genes regulated by other factors, most likely oxygen or carbon dioxide.

It was expected that the synthesis of proteins that regulate reproductive function and immune response would be altered in gravid *S. solidus* compared to infective plerocercoids obtained from the fish body cavity. Among the heat-induced proteins that are potentially involved in the reproduction process in *S. solidus*, mitogen-activated protein kinase A0A0X3PWN3 can be noted, which is one of the key enzymes of oocyte meiosis and fertilisation (Fan and Sun, 2004). Many of *S. solidus* protein kinases and protein phosphatases activated by heat can also participate in gametogenesis and impregnation in gravid worms by coordinating the activity of A0A0X3PWN3 (Table S7). Further studies of the localisation of these proteins in adult worms are necessary to clarify their significance for the reproduction of cestodes.

At the proteomic level, we did not observe a significant parasitic immune evasion of adult *S. solidus* compared to infective plerocercoids. Only 12 proteins upregulated at high temperature were KEGG annotated as potentially involved in the regulation of the immune system. Of these, seven are nonspecific regulators of signal transduction (protein kinases A0A0X3PXQ5, A0A0X3PWN3, A0A0X3PTZ8, phosphatase A0A0X3NTL5, Ras proteins A0A183SMP0, A0A0X3Q3W4 and 14-3-3 protein A0A0V0J2Z1), two are involved in protein folding (calnexin A0A0X3NKP9 and peptidyl-prolyl cis-trans isomerase A0A3P7CFD4), and two are associated with cell motility and adhesion (actin A0A0X3PZ06 and vinculin A0A0X3P131). Only the immunomodulatory cathepsin B-like peptidase A0A183SQT2 had a signal peptide for secretion and, more likely, could be involved in the regulation of definitive host immunity. This protein has been found in excretory/secretory products of *Echinococcus multilocularis* and gut extracts of parasitic nematode, and has been shown to degrade host connective tissue, suggesting an important role in helminth digestion and invasion (Sako et al., 2011; Long et al., 2015; Caffrey et al., 2018). Since adult *S. solidus* have impaired nutrition (Hopkins, 1950) and do not penetrate the avian intestinal wall, it can be assumed that the main reason for the expression of this protein in adult worms is the ability to destroy host immunoglobulins and components of the complement (Sako et al., 2011). To summarise, it seems that the anti-immune response of the parasite in the definitive host is not controlled by temperature, but the possibility of inducing parasite defence by direct interaction with host immune cells remains questionable.

### Conclusions

In this study, we analysed the spectra of *S. solidus* proteins to explore the molecular adaptations of parasites during trophic transmission from cold-blooded fish to warm-blooded definitive hosts. To identify the initial events that occur in a warm-blooded host, we studied changes in the spectrum of *S. solidus* proteins in the first hour after heating and found structural rearrangements of the parasite tegument at the molecular level. The long-term changes in a warm-blooded host are primarily associated with the consumption of energy stored by the larvae in the form of glycogen for protein biosynthesis. The observed activation of amino acids metabolism and protein production pathways in gravid worms presumably enable gametogenesis and egg production. Comparison of the obtained data with the results of transcriptomic studies confirmed the previously described changes in *S. solidus* gene expression in the definitive host, as well as revealed new markers of certain stages of worm development. We believe that further studies of the localisation of stage-specific helminth proteins will clarify their role in maintaining the parasitic lifestyle and in the relationship with hosts.

## MATERIALS AND METHODS

### Animal collection

Individuals of the three-spined stickleback *G. aculeatus* infected with *S. solidus* were caught with an aquarium net in August 2018 and 2019 in Lake Mashinnoe (North-West of Russia, 66°17′46.5″N 33°21′59.3″E). Infected fish were easily distinguished by their behaviour (swimming near the water surface) and a swollen abdomen. Live fish were delivered to the laboratory in barrels with water (with aeration) at 20-22°C and kept under such conditions until the experiment (no more than 5 days).

### Incubation of *S. solidus* plerocercoids

#### Short-term heating (1 h)

Three-spined sticklebacks were sacrificed by pithing (destroying the brain with a needle). The fish were then individually tightly wrapped with cling film to prevent the plerocercoids from crawling out of the host's body cavity and contacting the environment during incubation. Thereafter, the infected sticklebacks were immediately placed in water heated to 40°C for 1 h (Fig. 1). At the end of incubation, the fish were quickly taken out from the water, *S. solidus* were removed from the host body cavity (*n*=3), weighed, and frozen in liquid nitrogen. Plerocercoids (*n*=3) taken out from the fish body cavity immediately after the sacrifice of sticklebacks kept at 22°C were used as a control group. The intensity of infection of the studied fish was one plerocercoid per fish (Table S1). This experiment was repeated twice, in 2018 and 2019.

#### Long-term heating (48 h, heat-induced maturation)

Ten infected sticklebacks were sacrificed by pithing, while preventing pressure on the abdomen of the fish. The worms were removed from the fish body cavity and washed twice in a solution containing RPMI-1640 medium (Sigma-Aldrich) with 1% antibiotic antimycotic solution (Sigma-Aldrich). The intensity of infection of the studied fish was one plerocercoid per fish (Table S1). Next, the worms were placed in culture flasks with culture medium (RPMI-1640 medium, 0.1% antibiotic antimycotic solution and 10% glucose) and incubated in the water jacketed incubator (SHEL LAB) at 40°C (*n*=5) and 20-22°C (*n*=5) in a 5-10% CO_2_ atmosphere.

Egg production was assessed twice a day visually. In the treatment group the parasites became mature within 48 h, as determined by the presence of eggs in the medium. After this, the helminths from both groups were removed from the culture flasks, washed in fresh culture medium, frozen in liquid nitrogen and stored until proteome analysis. Plerocercoids (*n*=3) taken out from the fish body cavity immediately after the sacrifice of sticklebacks kept at 22°C were used as a control group. The whole long-term experiment was carried out in 2019.

### Sample preparation and LC-MS/MS analysis of *S. solidus* proteins

Frozen helminths (0.1-0.3 g) were ground with a pestle in a mortar with liquid nitrogen to a powder with the addition of 0.1 mol/l Tris-HCl (pH 7.6) with 1% protease inhibitor cocktail (Amresko) and 1% phenylmethanesulfonyl fluoride (Sigma-Aldrich). After thawing the extraction buffer, protein was precipitated with 100% trichloroacetic acid (final concentration 10%) (Sigma-Aldrich). After centrifugation at 12,000 ***g*** for 5 min, the pellet was washed with ice-cold 80% ethanol and ice-cold acetone by successive centrifugation–resuspension cycles. The final samples were lyophilised (Labconco FreeZone 6L) and stored until analysis at −80°C.

The lyophilisates were resuspended in the extraction buffer (300 μl) containing 4% sodium dodecyl sulfate (SDS) (Sigma-Aldrich) and 0.1 mol/l 1,4-dithiothreitol (Roche) in 0.1 mol/l Tris-HCl (pH 7.6). The total protein content in samples was measured according to the BCA method (Walker, 1994). A total protein amount of 100 mg for each sample was used for tryptic digestion according to the common FASP protocol (Wiśniewski et al., 2009). Briefly, protein disulfide bridges were reduced with 100 mmol/l 1,4-dithiothreitol in 100 mmol/l Tris-HCl (pH 8.5), and alkylation of thiols was performed with 55 mmol/l iodoacetamide (Sigma-Aldrich) in 8 mol/l urea in 100 mM Tris-HCl (pH 8.5). Detergents in the samples were exchanged with 100 mmol/l Tris-HCl (pH 8.5) using Microcon filters (10 kDa cut-off, Millipore). Tryptic digestion was carried out overnight at 37°C with trypsin (Sequencing Grade Modified, Promega) to protein ratio of 1:100 in a 50 mM tetraethylammonium bicarbonate (pH 8.5). To obtain the peptide solution, the filter samples were centrifuged at 11,000 ***g*** for 15 min at 20°C. The filters were then washed with 50 ml of 30% formic acid solution (Sigma-Aldrich) by centrifugation at 11,000 ***g*** for 15 min in a thermostatic centrifuge at 20°C. The filtrates were dried in a vacuum concentrator and dissolved in 20 ml of 5% formic acid for subsequent LC-MS analysis.

The separation of peptide mixture was performed using an Ultimate 3000 RSLCnano chromatographic HPLC system (Thermo Fisher Scientific, MA, USA) connected to a Q-exactive HFX mass spectrometer (Thermo Fisher Scientific). One microgram of peptides in a volume of 1-4 µl was loaded onto the Acclaim µ-Precolumn (0.5 mm х 3 mm, 5 µm particle size, Thermo Fisher Scientific) at a flow rate of 10 µl/min for 4 min in an isocratic mode of Mobile Phase C [2% acetonitrile (Sigma-Aldrich), 0.1% formic acid]. Then the peptides were separated with high-performance liquid chromatography (HPLC, Ultimate 3000 Nano LC System, Thermo Fisher Scientific, Rockwell, IL, USA) in a 15 cm long C18 column (Acclaim^®^ PepMap™ RSLC, inner diameter of 75 μm, Thermo Fisher Scientific, Rockwell, IL, USA). The peptides were eluted with a gradient of buffer B (80% acetonitrile, 0.1% formic acid) at a flow rate of 0.3 μl/min. The total run time was 90 min, which included initial 4 min of column equilibration to buffer A (0.1% formic acid), then gradient from 5 to 35% of buffer B over 65 min, then 6 min to reach 99% of buffer B, flushing 10 min with 99% of buffer B and 5 min re-equilibration to buffer A.

MS analysis of the samples was performed at least in triplicate with a Q Exactive HF-X mass spectrometer (Q Exactive HF-X Hybrid Quadrupole-OrbitrapTM Mass spectrometer, Thermo Fisher Scientific). The temperature of the capillary was 240°C, and the voltage at the emitter was 2.1 kV. Mass spectra were acquired at a resolution of 120000 (MS) in a range of 300-1500 m*/z*. Tandem mass spectra of fragments were acquired at a resolution of 15000 (MS/MS) in the range from 100 m*/z* to m/*z* value determined by a charge state of the precursor, but no more than 2000 m*/z*. The maximum integration time was 50 ms and 110 ms for precursor and fragment ions, respectively. AGC target for precursor and fragment ions were set to 1×10^6^ and 2×10^5^, respectively. An isolation intensity threshold of 50,000 counts was determined for precursor selection, and up to 20 top precursors were chosen for fragmentation with high-energy collisional dissociation (HCD) at 29 NCE. Precursors with a charge state of +1 and more than +5 were rejected, and all measured precursors were dynamically excluded from triggering a subsequent MS/MS for 70 s.

### Protein quantification and functional annotation

The mass spectra raw files were loaded into the MaxQuant v.1.6.4.3 program (Cox and Mann, 2008). The searches were performed using the Andromeda algorithm (built into MaxQuant) using the *Schistocephalus solidus* database loaded from UniProt/Swiss-Prot database (Proteome IDs UP000275846 and UP000050788 (Fontenla et al., 2017; International Helminth, 2019, accessed in May 2020; 43048 sequences). The following search parameters were set: digestion enzyme was trypsin with a maximum of two missed cleavages; 5.0 ppm as MS1 and 0.01 Da as MS2 tolerances; fixed modification: carbamidomethylation (Cys); variable modifications: N-terminal proteins acetylation and methionine oxidation (Met). Peptide Spectrum Matches (PSMs), peptides and proteins were validated at a 1% false discovery rate estimated using the decoy hit distribution.

Protein quantification was based on the label-free quantification (LFQ) method. The data matrix resulting from the MaxQuant analysis was loaded into Perseus software v. 1.6.2.3. The data were filtered to exclude proteins identified by modified (only identified by side) and reverse peptides, potential contaminants and proteins with fewer than two unique peptides. Obtained LFQ intensities are available in Table S2. An imputation of missing values procedure was performed for proteins that had LFQ-intensities for two from three technical repeats (Välikangas et al., 2018). Imputation of the missing values was done using the bpca method in the PCAmethod package for RStudio (Stacklies et al., 2007). The obtained data matrix of averaged replicates can be found in Table S3.

The functional annotation of identified *S. solidus* proteins was analysed using the Blast2GO, InterPro, BlastKOALA and QuickGO services (Götz et al., 2008; Binns et al., 2009; Kanehisa et al., 2016; Mitchell et al., 2019). Visualisation of KEGG pathway enrichment integrated with lipid enrichment was performed using the “Pathview” package (Luo and Brouwer, 2013) for the R computing environment. GO-term enrichment was analysed using the “topGO” package for R (Alexa and Rahnenfuhrer, 2021).

The presence of transmembrane regions was predicted using the TMHMM algorithm (version 2.0) and Phobius (Krogh et al., 2001; Brown et al., 2012). Signal peptides of classically and non-classically secreted proteins were predicted using SecretomeP 2.0 and SignalP 5.0 services (Bendtsen et al., 2004; Almagro Armenteros et al., 2019).

The unique *S. solidus* protein sequences were filtered from *S. solidus* protein database loaded from UniProt/Swiss-Prot (accessed in May 2020; 43048 sequence identifiers) using the CD-HIT Suite service with 100% sequence identity cut-off settings (Huang et al., 2010). The resulting groups, consisting of synonymous names of protein sequences, were used to compare the expression of *S. solidus* proteins with the previously described transcriptomic (Hébert et al., 2017) and other published data (Tables S4 and S5).

### Statistical analysis

The comparison of protein expression in the control and experimental groups was performed using the reproducibility-optimised test statistic in the ROTS package for R (R Core Team, 2017), which optimises the choice among a family of modified t-statistics (Suomi et al., 2017). Before the analysis, the LFQ-intensities were normalised using the normalizeVSN function in the limma package (Ritchie et al., 2015).

### Ethical approval

All animal experiments were in accordance with the EU Directive 2010/63/EU for animal experiments and the Declaration of Helsinki and were approved by the Animal Subjects Research Committee of the Institute of Biology at Irkutsk State University (Protocol 5, 16 April 2018).

## Supplementary Material

Supplementary information
