# Supplementary material for: Temperature-induced reorganisation of Schistocephalus solidus (Cestoda) proteome during the transition to the warm-blooded host
Source: Biol Open. 2021 Nov 17;10(11):bio058719. doi: 10.1242/bio.058719 (PMC8609239; doi:10.1242/bio.058719)
Supplement: Supplementary information [file biolopen-10-058719-s1.pdf]

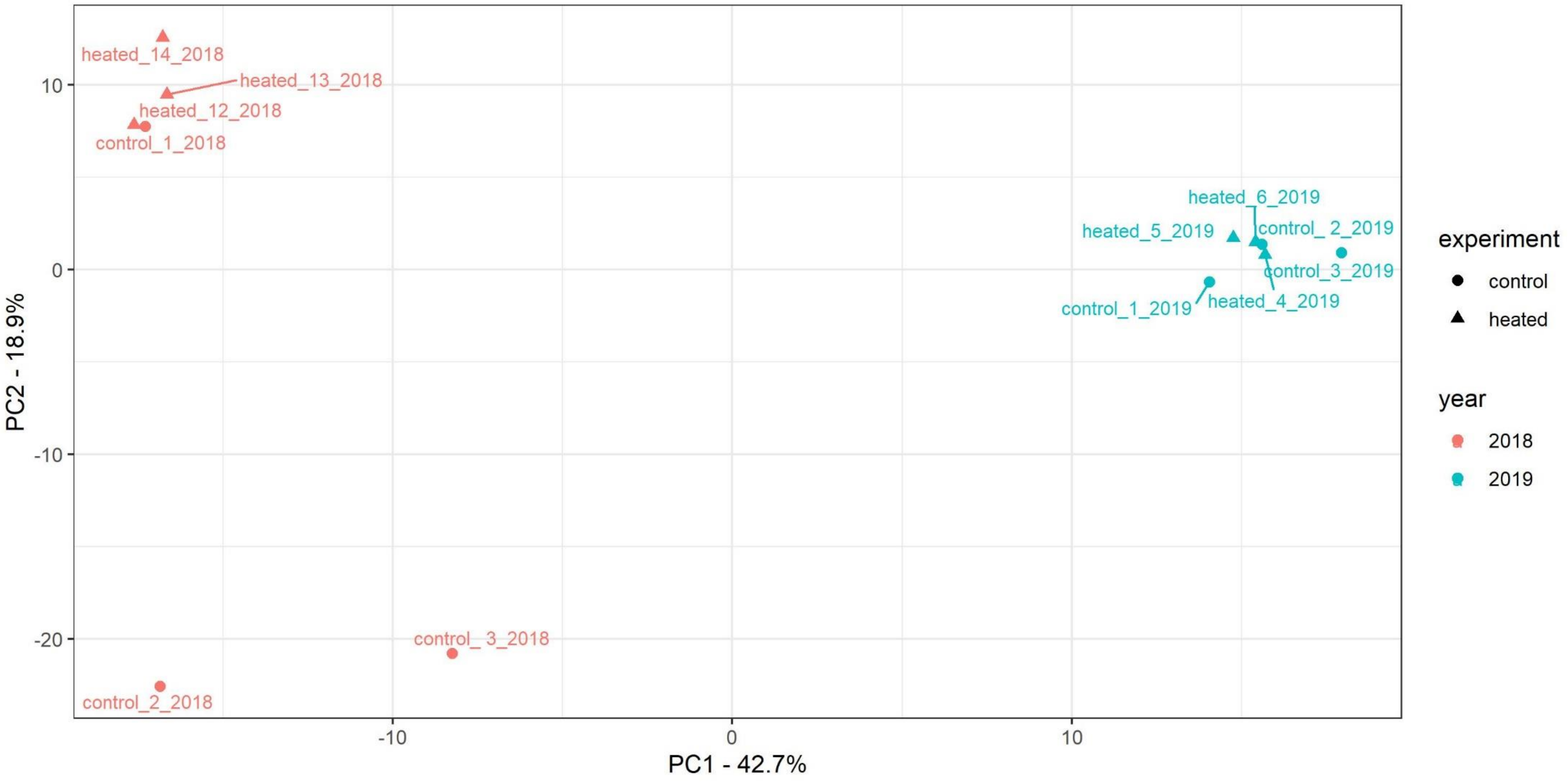

Fig. S1. Principal component analysis of content of proteins of *S. solidus* individuals in short-term experiments.

Biology Open • Supplementary information

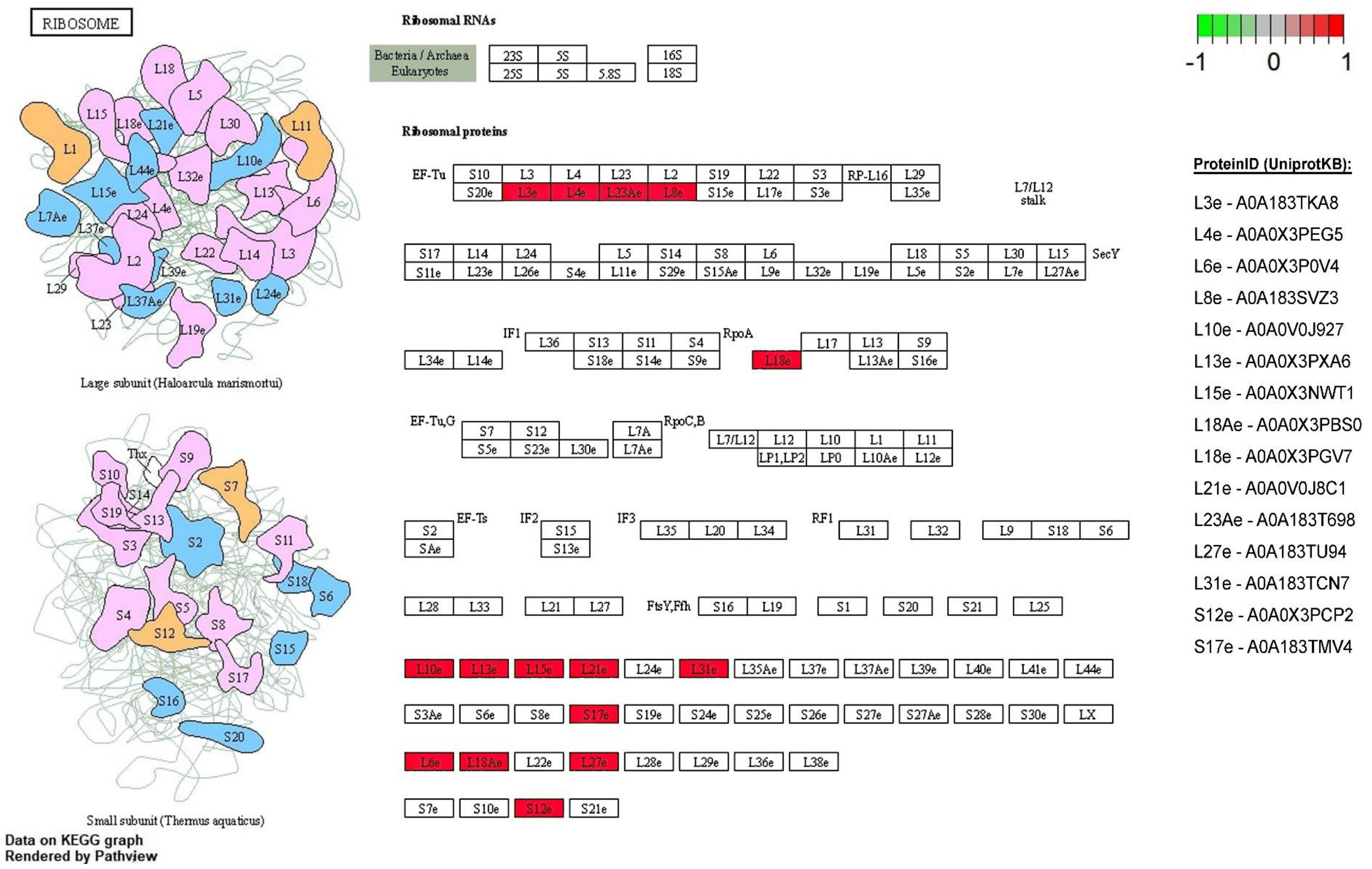

Fig. S2. Enrichment of Ribosome KEGG Pathway (K03010) among “heat-related” proteins in adult *S. solidus*.

Biology Open • Supplementary information

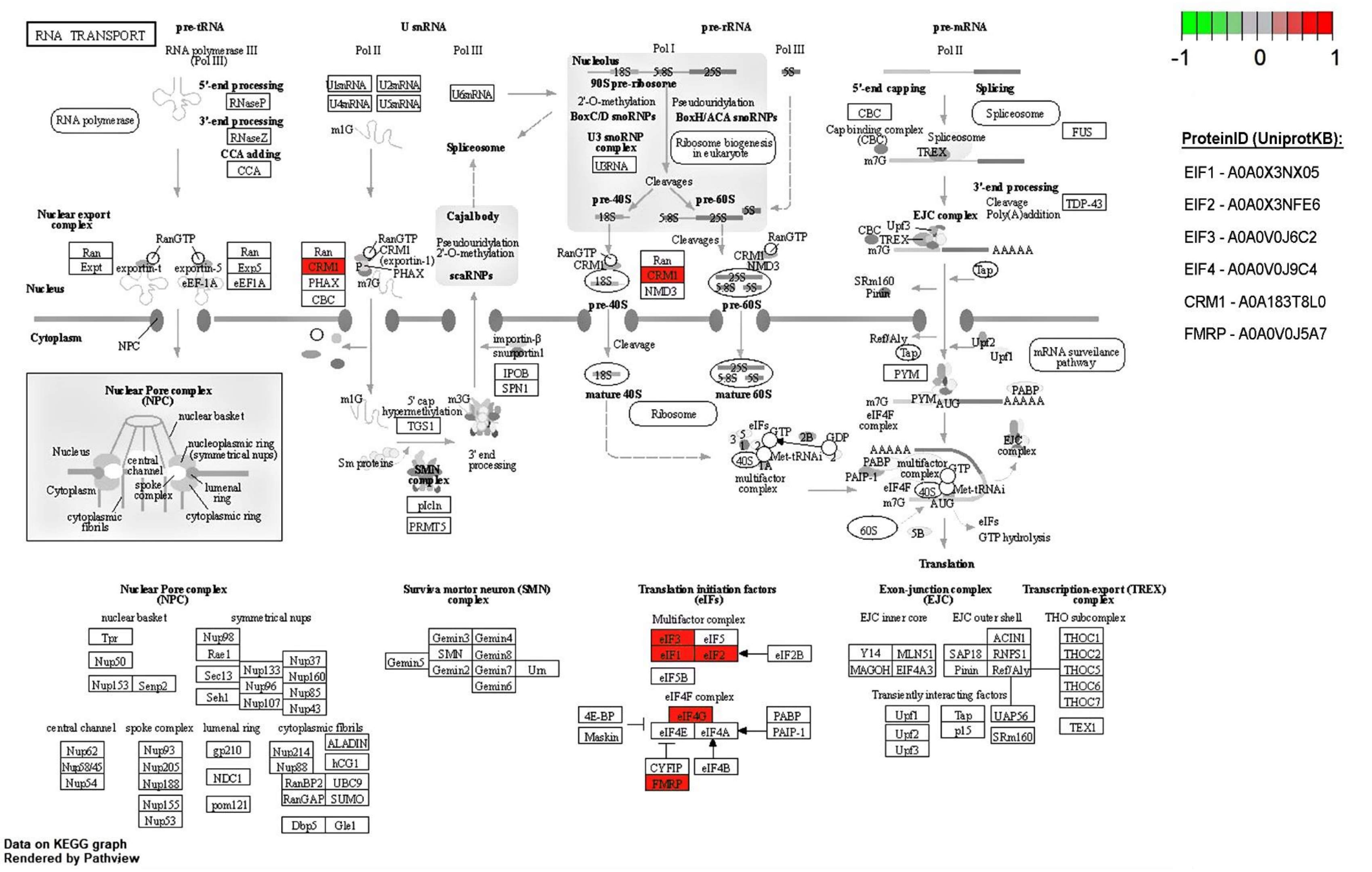

Fig. S3. Enrichment of RNA transport KEGG Pathway (K03013) among “heat-related” proteins in adult *S. solidus.*

Biology Open • Supplementary information

Table S1. Description of samples

Click here to download Table S1

Table S2. LFQ intensity of identified S. solidus proteins (raw data)*

Click here to download Table S2

Table S3. LFQ intensity of identified S. solidus proteins, imputed by bpca method, filtered and averaged

Click here to download Table S3

Table S4. Differently expressed proteins (DE) in Schistocephalus solidus infective plerocercoids obtained from fish and incubated at 40 °C for 1 hour

Click here to download Table S4

Table S5. Differentially expressed proteins (DE) in Schistocephalus solidus infective plerocercoids and in adult worms incubated at 40°C for 48 hours according to the proteome (our data) and transcriptome (Hébert et al., 2017*) studies.

Click here to download Table S5

Table S6. Results of functional GO terms* enrichment analysis performed by topGO package

Click here to download Table S6

Table S7. KEGG annotation* of differentially expressed proteins in Schistocephalus solidus infective plerocercoids and in adult worms.

Click here to download Table S7
